# Mate choice and genetic monogamy in a biparental, colonial fish

**DOI:** 10.1093/beheco/arv011

**Published:** 2015-03-05

**Authors:** Franziska C. Schaedelin, Wouter F.D. van Dongen, Richard H. Wagner

**Affiliations:** Konrad Lorenz Institute of Ethology, Department of Integrative Biology and Evolution, University of Veterinary Medicine Vienna, Savoyenstrasse 1a, 1160 Vienna, Austria

**Keywords:** assortative mating, cichlid, colony, extrapair paternity.

## Abstract

In socially monogamous species, in which both sexes provide essential parental care, males as well as females are expected to be choosy. Whereas hundreds of studies have examined monogamy in biparental birds, only several such studies exist in fish. We examined mate choice in the biparental, colonial cichlid fish *Neolamprologus caudopunctatus* in Lake Tanganyika, Zambia. We genotyped more than 350 individuals at 11 microsatellite loci to investigate their mating system. We found no extrapair paternity, identifying this biparental fish as genetically monogamous. Breeders paired randomly according to their genetic similarity, suggesting a lack of selection against inbreeding avoidance. We further found that breeders paired assortatively by body size, a criterion of quality in fish, suggesting mutual mate choice. In a subsequent mate preference test in an aquarium setup, females showed a strong preference for male size by laying eggs near the larger of 2 males in 13 of 14 trials.

## Lay Summary

We report a rare case of genetic monogamy in a biparental fish species. Males and females paired assortatively by size, which is compatible with mutual mate choice. Mate choice in monogamous species is interesting because both sexes provide essential parental care, making males, as well as females, choosy. Social monogamy in the form of biparental care is well known from a variety of species, but uncommon in fish.

## INTRODUCTION

Mate choice affects fitness in all sexually reproducing animals. Individual variation in mating success produces sexual selection of varying intensities. Trivers (1972) provided the theoretical framework for studying mate choice in relation to parental investment. In species in which males do not provide parental care, sexual selection is intense in that male mating success is highly skewed, such as in leks in which only one or several males obtain matings from numerous females (Höglund and Alatalo 1995). In contrast, sexual selection is weaker in genetically monogamous species in which males pair with 1 female and typically provide critical parental care (Andersson 1994). Parental care by monogamous males selects for males, as well as females, to be choosy (e.g., Jones and Hunter 1993). Mutual mate choice for higher quality mates, often accompanied by intrasexual competition, frequently leads to assortative mating by some criteria of individual quality (Crespi 1989; Harari et al. 1999). Monogamy constrains female mate choice in that only one female can pair with the top male in a population (Gowaty and Bridges 1991). Monogamy also constrains the ability of males to fertilize more than 1 female, leading to mixed mating strategies where males attempt to fertilize the mates of other males that then care for the unrelated offspring. For biparental males, the fitness benefits of fertilizing additional females are apparent, as are the costs of losing paternity. Hence, there has been long-term interest in paternity assurance tactics, especially in mate guarding in which males maintain close surveillance of their fertile mates to block extrapair matings (Birkhead and Møller 1992). An increased risk of brood desertion in cases of increased uncertainty of paternity can also constrain the pursuit of extrapair matings (Mulder et al. 1994; Birkhead and Møller 1996; Gowaty 1996). Alternatively, extrapair fertilizations (EPFs) may be constrained by asynchronous breeding, which results in a lack of neighboring potential extrapair mates (Stutchbury and Morton 1995). In contrast, females do not typically produce more offspring after mating with multiple males. Therefore, it was generally assumed that females spawning in the presence of sneaker males were not attempting to obtain EPFs. However, Reichard et al. (2007) show that females of at least 3 unrelated externally fertilizing fish species do prefer to mate in the presence of sneaker males. The females may obtain genetic benefits from EPFs such as “good genes” from males of higher quality than their social mate (Hunter et al. 1993) or by obtaining fertilizations from extrapair males that are more genetically dissimilar from them than their mates in order to prevent inbreeding (Blomqvist et al. 2002) or increase genetic variability in the offspring (Tregenza and Wedell 2000; Landry et al. 2001; Blomqvist et al. 2002; Freeman-Gallant et al. 2003; Olsson et al. 2003; Thuman and Griffith 2005).

Extrapair mating is a strategy that has been predominately studied in birds, in which approximately 90% of species breed in biparental pairs (Lack 1968). The advent of genetic methods for identifying parentage led to an explosion of studies revealing EPFs in hundreds of socially monogamous birds (Westneat et al. 1990; Griffith et al. 2002) and in 17 of 22 investigated species of mammals (Cohas and Allainé 2009), in which biparental care is uncommon (Kleiman and Malcolm 1981; Royle et al. 2012). Biparental care in fishes is the least common form of parental care (Mank et al. 2005), and to our knowledge, only 9 parentage studies have been reported in such species (Coleman and Jones 2011; Takahashi et al. 2012; Langen et al. 2013), of which EPFs were observed in 4 species (Coleman and Jones 2011).

Here, we investigate multiple facets of mate choice in *Neolamprologus caudopunctatus*, a biparental, colonial fish endemic to Lake Tanganyika in southern Africa. We used microsatellite genotyping to examine the potential occurrence of EPFs and to evaluate whether there is evidence of mate preference for genetically dissimilar mates. We also combined field data and a preference test to determine whether individuals select mates according to their body size, a general criterion of quality in fish because larger individuals of both sexes are more effective in territorial defense (Barlow et al. 1986; Rowland 1989; Koops and Grant 1993) and larger females are more fecund (Wootton 1984; Kolm 2002).

## METHODS

### Study species

*Neolamprologus caudopunctatus* (adult total length 4.5–6cm) is a colonial cichlid endemic to the southern part of Lake Tanganyika. This sexually monomorphic substrate breeder inhabits the rocky–sandy shore at depths of several to more than 25 m. Nonreproducing adults form large midwater shoals, whereas breeding pairs occupy the substrate where they construct breeding cavities by removing sand with their mouths from under stones (Ochi and Yanagisawa 1999). Both parents defend breeding cavities containing eggs and larvae and subsequently free-swimming fry for about 40 days until they achieve independence (Ochi and Yanagisawa 1999).

### Fieldwork

**Figure 1:**
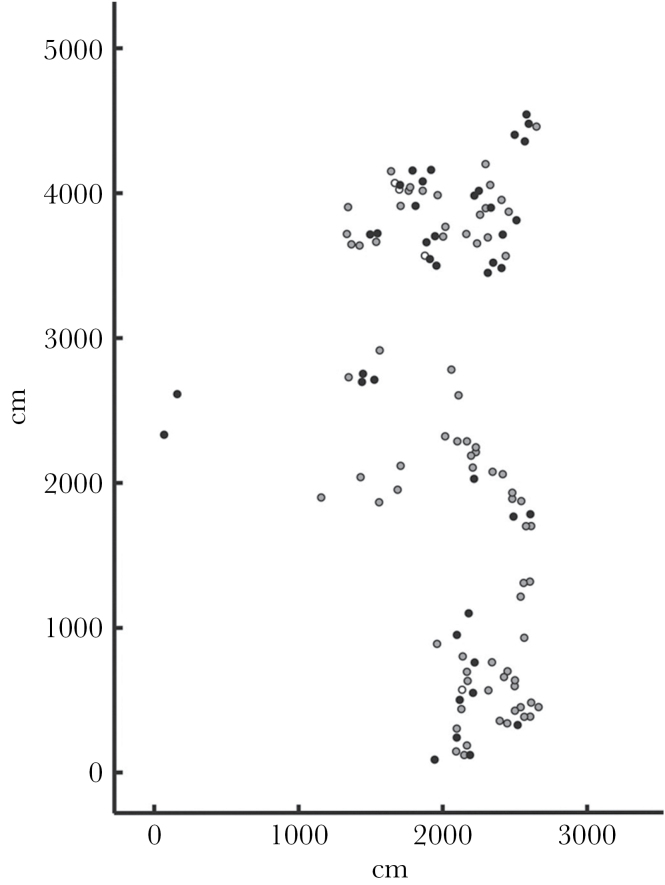
Map of all nests of pairs of *Neolamprologus caudopunctatus* in the study colony at Mpulungu Bay. Black dots indicate nests where paternity analysis was carried out, and open and grey dots indicate nests of pairs where no genetic sampling was done.

The study was conducted on the southern shore of Lake Tanganyika, northwest of Mpulungu, Zambia, in Kasakalawe Bay (08°46′46.6″S/31°04′44.4″E). In October and November 2005, we studied a colony of *N. caudopunctatus* breeding at a depth of 12–14 m, occupying an area of approximately 50×30 m. We numbered all 118 breeding cavities defended by a pair by SCUBA (self-contained underwater breathing apparatus) diving (Figure 1). To map the whole colony underwater, we laid out 2 orthogonal axes out of sisal string and measured nest distances to both axes with measuring tape. We used PVC sheet and pencil to note our measurements. For genetic analyses, we used monofilament nets to capture breeding pairs, measured their total body length, including the tail, and collected fin clippings from the dorsal fin in situ. We clipped 1.0–1.4cm off the middle of the dorsal fin of females and the end of the dorsal fin of males for sex identification. All adults were subsequently released. We then sacrificed the offspring by spraying an overdose of clove oil into the breeding cavity of the pair and collected the offspring in Eppendorf tubes. On shore, we transferred samples in tubes filled with 97% ethanol. We only sampled families with free-swimming fry.

### Size preference test

We carried out a mate preference test in aquariums at the Konrad Lorenz Institute of Ethology to determine whether females preferred larger males. We created the setup by dividing a 160-L tank into 3 compartments with 2 mesh partitions (mesh size 3.3mm). We then introduced potential nesting shelters in the middle compartment in the form of 2 small PVC slates (10×15cm) leaned against each mesh partition. A male was introduced into each of the 2 side compartments and a female in the middle compartment, wherein no individual fish was used more than once. This provided the female with access to both potential nesting sites. Males in the side compartments could potentially detect visual and olfactory cues and fertilize eggs through the mesh but could not physically interact with each other or the female in the central compartment. Our institute’s fish population originated from the southern tip of Lake Tanganyika and comprises wild caught individuals and their first-generation offspring. All tanks were supplied with a 5-cm layer of sand and a water filter. The light cycle was 12-h light and 12-h darkness. The fish were fed with commercial flake food once a day 4 times per week and with frozen food the other 3 days of the week.

In each trial, we chose one male to be 0.4cm longer in standard length (from the tip of the longest jaw to the end of the base of the caudal fin) than the other male. The compartment of the larger male was randomly selected. Sizes of the larger males ranged from 6.0 to 6.6cm and for the smaller males from 5.4 to 6.1cm. Female body sizes ranged from 78.6% to 88.3% of the larger male. We checked nest sites daily for egg laying. We started to perform 21 trials, of which we excluded 4 trials because of sickness or death of 1 of the 3 fish included in each trial. After 22 days, we observed whether the female had produced eggs, and if so, whether on the side of the larger or the smaller male.

### Laboratory analyses

All adults and fry were genotyped at 11 microsatellite loci with 3–30 different alleles per locus and observed heterozygosities ranging from 0.16 to 1.00. Detailed information can be found in Schaedelin et al. (2013). Fragment sizes were estimated using the Beckman Coulter CEQ 8000 fragment analysis software or ABI Genemapper 4.0.

### Genetic data analysis

We implemented CERVUS 3.0 (Kalinowski et al. 2007) based on 61 adults to characterize the loci and to conduct parentage analyses. When we found a mismatch at only one locus between a fry and its putative parents, we repeated the polymerase chain reaction to confirm the mismatch. The 11 loci resulted in a high probability of identifying parent pairs of the offspring (exclusion probability of second parent: 0.9999; exclusion probability of parent pair: 0.999999). Parentage was assigned using both strict (95%) and relaxed confidence (80%) at the population level. To determine the critical likelihood score for these confidence levels, we conducted a parentage simulation (parent pairs of known sex). The criteria used for the simulation were 100 000 simulated offspring, a sampling efficiency of 28% for both males and females (66 adults of 236 permanently present within the colony at the time of the study), 99.8% of loci typed, and a mistyping error rate of 0.01. Offspring with one or fewer mismatches with each parent were assigned as within-pair young, thus reducing the probability of incorrectly assigning an offspring as extrapair due to a genotyping error or mutation (Jones et al. 2010).

We estimated genetic similarity following Mathieu et al. (1990) to explore mating patterns between the sexes. Here, genetic similarity was estimated as the probability that a given pair will produce homozygous offspring (Phm). For each locus (*l*), Phm is equal to:

$${\text{Phm}}_{xy}(l)=\frac{s_{\text{ac}}+s_{\text{ad}}+s_{\text{bc}}+s_{\text{bd}}}{4},$$

where *s*_*ij*_ equals 1 if alleles *i* and *j* are the same and 0 otherwise. Across all loci, a weighted average is used

$${\text{Phm}}_{xy}=\frac{\sum_{l}\frac{1}{p_{l}}{\text{Phm}}_{xy}(l)}{\sum_{l}\frac{1}{p_{l}}},$$

where *p*_*l*_ is the probability of an individual being homozygous by chance at locus *l*. The probability that an individual will be homozygous by chance at one locus equals 1 − observed heterozygosity. Phm_*xy*_ will be close to 1 for more genetically similar individuals, who are thus more likely to produce homozygous offspring. Belkhir et al. (2002) showed that this index of genetic similarity is a superior alternative compared with other measurements of genetic similarity (e.g., Queller and Goodnight 1989; Lynch and Ritland 1999) when the number of loci used is relatively low. Phm_*xy*_ was calculated for all potential pairings between males and females sampled (30 males and 31 females, resulting in 930 potential pairings) using IDENTIX software (Belkhir et al. 2002). To test whether pairings occur between more genetically dissimilar individuals than expected by random, we first compared the mean Phm_*xy*_ for all observed pairings with that of all remaining potential pairings within the population. Second, we tested whether observed pairs with low Phm_*xy*_ values were more frequent and whether observed pairs with high Phm_*xy*_ values were less frequent than expected by chance. This was done by calculating the first quartile of Phm_*xy*_ values in observed pairs (Phm_*xy*_ = 0.254) and comparing the frequency of pairs that had Phm_*xy*_ values below this value of the total 29 observed and total 29 randomly created via a chi-square test. The upper quartile was calculated for randomly created pairs (Phm_*xy*_ = 0.422), which was then used to compare the frequency of observed and random pairs with high Phm_*xy*_ values.

### Statistics

All statistical analyses were carried out with IBM SPSS Statistics 20. Data were tested for normality with the Shapiro–Wilk test. We used Pearson correlations for normally distributed data and Spearman rank correlations for nonnormal data.

## RESULTS

### Extrapair parentage

All 118 nesting sites in the study colony were numbered during a 1-week marking period. During multiple repeated visits over 2 months, we examined the contents of 96 nests, of which 43 (45%) contained free-swimming fry. We genotyped 291 fry from 32 families, of which we obtained DNA samples from both putative parents in 29 families and 1 in 3 families (1 male parent and 2 female parents). No cases of EPFs were found in any of the 32 families. Parentage was assigned to 217 fry (74.6%, *N* = 291) with strict confidence (including 1 mismatch for 11 fry) and a further 33 (11.3%) with relaxed confidence (including 1 mismatch for 2 fry). Fifty-nine percent of nests contained fry that did not match either parent and, as we have reported, were apparent cases of adoption (Schaedelin et al. 2013). Only 13 of 291 fry had 1 mismatch; however, mismatches were evenly distributed in that they occurred with the female in 7 cases and 6 cases with the male. Therefore, the maximum paternity loss frequency would be 2.1%. Mismatches are, therefore, highly unlikely to represent cases of extrapair paternity.

### Mate choice for size

**Figure 2:**
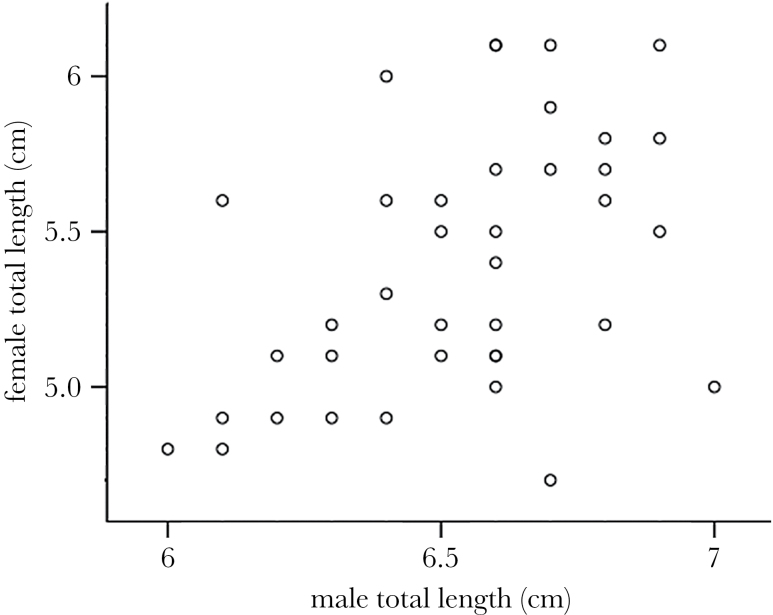
Male body size versus female body size within pairs. Body size was measured as total length in centimeters.

There was a positive correlation between male and female body size within pairs breeding in Lake Tanganyika (Figure 2; total body length: *N* = 38; *r* = 0.475; *P* = 0.003). In our choice test in the aquarium setup, 13 of 14 females that had laid eggs did so on the side of the larger male (*N* = 14, 13:1, binominal test, *P* = 0.002).

### Mate choice and genetic similarity

**Figure 3:**
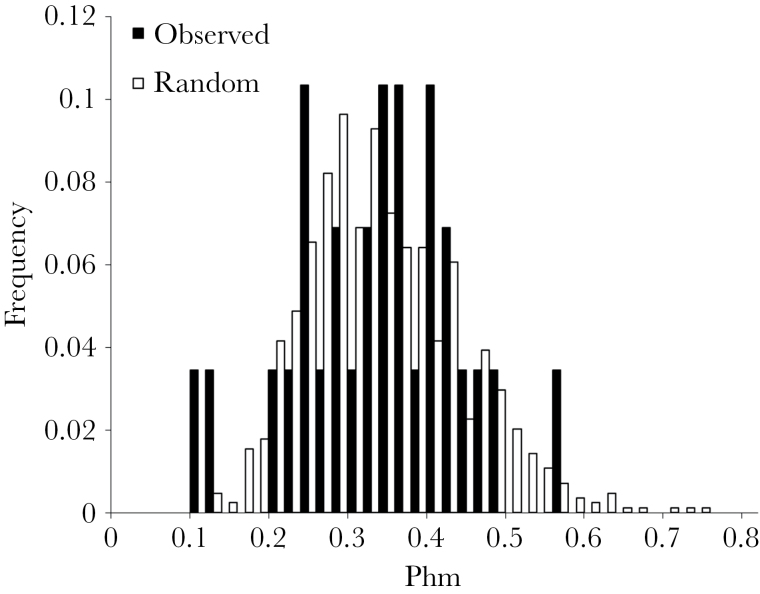
Frequency distribution of genetic similarity (estimated using the Phm index) for observed pairs within the population (black bars) and for all possible male–female dyads (white bars).

We found no significant difference in genetic similarity of mates versus an expected distribution from randomly generated pairings (Figure 3; mean genetic similarity of mates—log transformed: observed = 0.32±0.10 SD vs. random pairings = 0.32±0.10 SD; *F*_1,868_ = 0.254, *P* = 0.614). The frequency of observed pairs with low Phm_*xy*_ values was not greater than expected by chance (pairs with low Phm_*xy*_ values: observed = 8/29, random = 12/29; χ^2^ = 0.800, degrees of freedom [df] = 1, *P* = 0.371). Similarly, the frequency of observed pairs with high Phm_*xy*_ values was not lower than expected by chance (pairs with high Phm_*xy*_ values: observed = 4/29, random = 8/29; χ^2^ = 1.333, df = 1, *P* = 0.248).

## DISCUSSION

### Genetic monogamy

Our field and genetic data allowed us to examine several aspects of mate choice in a colonial, biparental fish. DNA analyses of *N. caudopunctatus* revealed no convincing cases of EPFs in nearly 300 offspring. Parental investment theory predicts a positive relationship between male care and paternity (Trivers 1972), which has been studied in a wide range of taxa, including fish. Some studies support the hypothesis that males reduce parental care with diminished confidence of paternity (Dixon et al. 1994; Weatherhead et al. 1994; Wagner et al. 1996; Neff and Gross 2001; Hunt and Simmons 2002), whereas other studies have not supported this idea (Jamieson et al. 1994; Westneat 1995; Freeman-Gallant 1997; Kempenaers et al. 1998). Some variation across species appears to be caused by differences in life histories (Kolm et al. 2006), especially the degree to which paternal care is crucial for successful reproduction (Barlow 1974; Kleiman 1977; Weatherhead 1990). We thus expect low frequencies of EPFs in species with high levels of paternal care. In fish taxa where males carry embryos internally, such as seahorses and pipefishes, no EPFs were found in any of 8 species studied (Coleman and Jones 2011). The absence of EPFs in this group may have evolved, and may be maintained, by their mode of spawning in which females deposit their eggs directly onto a male or into his internal pouch. Among male-caring fish species in which males do not carry the eggs, EPFs were found in 15 of 18 species, although usually at low frequencies (Coleman and Jones 2011).

Biparental brood care is uncommon in fishes, occurring in 10% of 51 families (Mank et al. 2005). Our findings comprise the 10th parentage study of a biparental species, in which 5 appear to be genetically monogamous. Genetic monogamy was found in the substrate breeder *Pelvicachromis taeniatus* (Langen et al. 2013), in the mouth brooding cichlids *Eretmodus cyanostictus* (Taylor et al. 2003) and *Xenotilapia rotundiventralis* (Takahashi et al. 2012) and possibly in the largemouth bass *Micropterus salmoides* in which only 1 of 24 broods appeared to be multiply sired (DeWoody et al. 2000).

The genetic monogamy of *N. caudopunctatus* might be at least partially explained by the particular combination of 3 features of their breeding system acting in unison (Schädelin et al. 2012) to minimize opportunities for extrapair spawning. First, nests are continuously guarded by both mates, who do not need to leave the vicinity of their breeding cave because they obtain their food of plankton in the water column near the nest. Continuous mate guarding is considered to be the most effective form of paternity assurance in birds (Birkhead and Møller 1992) and has been suggested to explain genetic monogamy in other taxa, for example, in the antelope Kirk’s dik dik *Madoqua kirkii* (Brotherton and Rhodes 1996).

Second, *N. caudopunctatus* pairs construct nesting caves with very narrow entrances to minimize intrusions by predators and conspecifics. Likewise in *P. taeniatus*, the only other biparental substrate breeding cichlid with confirmed genetic monogamy, Langen et al. (2013) attributed the absence of EPFs to continuous mate guarding and the narrow breeding cavity entrance to diminish the threat from sneakers. Ota et al. (2014) found that the smaller the nest opening of a *Lamprologus lemarii* nest was, the higher the siring success of the territorial male, possibly because it impedes the access of sneaker males to the nests. Genetic monogamy has also been attributed to the narrow entrances of well-sheltered breeding caves in the striped darter *Etheostoma virgatum* (Porter et al. 2002). Third, external fertilization prevents females from obtaining EPFs by visiting extrapair males, as occurs in birds. Hypothetically, EPFs could occur even with external fertilization in our study species if females were able to deliver fertilized eggs in their mouths to another nesting cave. This seems however unlikely, given our many hours of observation over several years without having witnessed such behavior.

Like *N. caudopunctatus*, *Variabilichromis moorii* and *Neolamprologus pulcher* are substrate brooding cichlids in which both mates defend their nest, yet both species show moderate to high frequencies of extrapair paternity (Dierkes et al. 1999, 2008; Heg et al. 2006, 2008, 2009; Heg and Hamilton 2008; Sefc et al. 2008; Stiver et al. 2009). This contrast might be explained by interspecific differences in breeding systems, which may allow extrapair offspring to provide certain benefits, such as in recruiting additional helpers at the nest, as suggested for *N. pulcher* (Heg et al. 2009; Zöttl et al. 2013) or in diluting offspring predation in the shoal, as found in *Cichlasoma citrinellum* (McKaye and McKaye 1977). Similarly, we have found a high frequency of apparent adoption in *N. caudopunctatus*, where 59% of broods contained fry unrelated to both parents (Schaedelin et al. 2013). This raises the question of how extrapair males can be excluded from nests while fry belonging to neither parent are common among broods. As we have suggested, parents may tolerate and even invite unrelated offspring to join their broods for the benefit of predator dilution (Schaedelin et al. 2013).

### Assortative mating

We found that *N. caudopunctatus* pair assortatively by body size in nature. This pattern also occurred in our institute’s huge 16 000-L ring-shaped aquarium in which multiple males and females were allowed to form pair bonds (Demus 2010). Our separate size preference test revealed that females preferred larger males by laying their eggs near the larger of 2 males. Given that territory size was kept constant in this test, body size alone may be considered a significant criterion of female mate choice in this species. In fish species generally, larger individuals are more effective in territorial defense, and thus, body size may serve as an indicator of defense ability (Barlow et al. 1986; Rowland 1989; Koops and Grant 1993). Thus, larger *N. caudopunctatus* males probably have a higher success rate in defending their offspring against predators. Likewise, males are expected to prefer larger females because in fish generally, larger females are more fecund (Wootton 1984; Kolm 2002). The choosiness of both sexes in biparental species results in mutual mate choice such that intrasexual competition for higher quality (larger) mates in both sexes may have contributed to the observed pattern of assortative mating in our species.

Our mate choice experiment shows that in the absence of female–female competition, all females within a range of sizes will spawn with a larger male. But given that in nature mainly larger females pair with larger males, it means that intrasexual competition is operating.

Assortative mating may also occur as a by-product of another factor such as breeding habitat choice, as was found in *E. cyanostictus* in which females preferred larger territories defended by larger males (Taborsky et al. 2009). This seems unlikely in *N. caudopunctatus*, given that in our 16 000-L aquarium, which resembles a natural spatial scale for 5–6cm fish, males and females were frequently observed forming bonds before prospecting for breeding sites (Demus 2010). Additionally, in Lake Tanganyika, we have observed many caves that were defended by pairs but never by individuals.

### Mate choice and relatedness

Our DNA data also allowed us to examine whether mate choice is influenced by genetic relatedness in *N. caudopunctatus*. Avoiding pairing with genetically similar mates can be an adaptive behavior to avoid deleterious effects of inbreeding on reproduction (Mulard et al. 2009). However, we found that breeders did not select mates on this basis, even when the extreme quartiles were compared. The apparent lack of inbreeding avoidance in our population may suggest another reason for the absence of EPFs, namely that selection may be weak or nonexistent for pursuing EPFs with genetically dissimilar mates.

## CONCLUSIONS

We found evidence of genetic monogamy in a biparental fish, only one of several such species to be so identified. This finding belongs to the broad context of parentage and parental care and contributes to the body of literature that shows markedly high variation in EPF frequency among fish species and other taxa. Although the lack of EPFs in a species with critical male care is expected, it is often not found. Our study adds to evidence that EPF frequencies may be significantly influenced by specific details of the life history and behavior of a given species or even population. As we suggest, the genetic monogamy of *N. caudopunctatus* might be explained by the confluence of several factors of their mating strategies and features of their environment. As more parentage studies are reported in fish, researchers will be able to evaluate the variation in EPF frequency among species in relation to the specific details of their life histories. This suggests the need for more such studies in fish to determine whether general patterns exist in line with theory.

We further found that monogamous pairs of *N. caudopunctatus* are size-assortatively mated, which is probably the outcome of preferences and intrasexual competition by both sexes for larger mates. Further tests may reveal whether larger pairs obtain higher fitness. Finally, by providing evidence that breeders do not select mates on the basis of genetic similarity or dissimilarity, our study also contributes to a growing literature on the genetic factors of mate choice.

## FUNDING

This project was funded by the Austrian Science Fund (FWF) grants P17468 and P20401 to R.H.W., by a travel grant from the Swiss Academy of Science to F.C.S. without which this field trip would not have been possible, and the Austrian Academy of Sciences.
